# Supplementary material for: Oxytocin administration enhances pleasantness and neural responses to gentle stroking but not moderate pressure social touch by increasing peripheral concentrations
Source: eLife. 2023 May 12;12:e85847. doi: 10.7554/eLife.85847 (PMC10229136; doi:10.7554/eLife.85847)
Supplement: Supplementary file 1. — Table 1A. Treatment effects on mood. Table 1B. Treatment effects on post-treatment plasma OT change (pg/ml). Table 1C. Confounding effects of individual perceived gender on behavioral pleasantness rating scores. Table 1D. Treatment effects on physiological indices. [file elife-85847-supp1.docx]

**Supplementary file 1**

**Supplementary Tables 1A through 1D**

**Supplementary file 1. Table 1A.** Treatment effects on mood

|  | **Intranasal OT** | **Oral OT** | **PLC** |  |  |  |
| --- | --- | --- | --- | --- | --- | --- |
|  | *M ± sd* | *M ± sd* | *M ± sd* | *F* | *Post-hoc Bonferroni corrected tests* | |
| Positive affect | 18.71 ± 0.80 | 19.63 ± 0.78 | 17.42 ± 0.78 | Time: F(3, 504) = 11.12^***^ | Pre-treatment < post-medium pressure massage (*p* = 0.03) | |
|  |  |  |  |  | Pre-treatment < post-gentle stroking touch (*p* < 0.001) | |
|  |  |  |  | Treatment: F(2,168) = 2.04 | - | |
|  |  |  |  | Time x treatment: F(6,504) = 1.40 | - | |
| Negative affect | 11.00 ± 0.37 | 11.31 ± 0.36 | 11.15 ± 0.36 | Time: F(3, 504) = 27.10^***^ | Post-treatment < post-medium pressure massage (*p* < 0.001) | |
|  |  |  |  |  | Post-treatment < post-gentle stroking touch (*p* < 0.001) | |
|  |  |  |  | Treatment: F(2,168) = 0.17 | - | |
|  |  |  |  | Time x treatment: F(6,504) = 0.58 | - | |

Table notes: We conducted repeated-measures ANOVAs with time (pre-treatment vs. post-treatment vs. post-medium pressure massage vs. post-gentle stroking touch) as within-subject factor and treatment (intranasal OT vs. oral OT vs. PLC) as between-subject factor were employed separately for the positive and negative mood scores. For the positive mood, results showed a significant main effect of time (F(3, 504) = 11.12, *p* < 0.001), with significant decrease of the positive mood from pre-treatment to post- pressure massage (*p* = 0.03) and post-gentle stroking touch (*p* < 0.001). For the negative mood, there was also a significant main effect of time (F(3, 504) = 27.10, *p* < 0.001), with significant decrease of negative mood from post-treatment to post- medium pressure massage (*p* < 0.001) and post-gentle stroking touch (*p* < 0.001). For both sub-scale scores, there were no significant main effects of treatment and interactions (*p*s > 0.16).

**Supplementary file 1. Table 1B.** Treatment effects on post-treatment plasma OT change (pg/ml)

|  | **Intranasal OT** (G1) | **Oral OT**  (G2) | **PLC**  (G3) |  |  |  | |
| --- | --- | --- | --- | --- | --- | --- | --- |
| *Plasma OT change (pg/ml)* | *M ± sd* | *M ± sd* | *M ± sd* | *Treatment effect* | *G1vs. G2* | *G1 vs. G3* | *G2 vs. G3* |
| Post-treatment | 4.49 ± 0.58 | 1.78 ± 0.29 | -0.31 ± 0.35 | F(2,162) = 33.42^***^ | *p* < 0.001 | *p* < 0.001 | *p* = 0.001 |

Table notes: Supplementary one-way ANOVA by excluding the extreme values of intranasal and oral OT groups (z scores > 3) revealed a stable significant main effect of treatment.

**Supplementary file 1. Table 1C.** Confounding effects of individual perceived gender on behavioral pleasantness rating scores

| ***Source*** | ***F*** | ***p*** | ***η_p_^2^*** |
| --- | --- | --- | --- |
| *Effects of perceived gender on pleasantness ratings* | | | |
| Perceived gender | F(1, 169) = 0.60 | 0.44 | 0.004 |
| Condition | F(1, 169) = 57.32 | < 0.001 | 0.25 |
| Perceived gender x condition | F(1, 169) = 0.22 | 0.64 | 0.001 |
| *Effects of oxytocin on perceived gender* | | | |
| χ^2^ = 1.63, *p* = 0.44 | | | |
| *Effects of oxytocin and perceived gender on pleasantness ratings* | | | |
| Treatment | F(2, 165) = 3.93 | 0.02 | 0.046 |
| Perceived gender | F(1, 165) = 0.83 | 0.36 | 0.005 |
| Condition | F(1, 165) = 59.33 | < 0.001 | 0.26 |
| Perceived gender x condition | F(1, 165) = 0.34 | 0.56 | 0.002 |
| Perceived gender x treatment | F(2, 165) = 0.27 | 0.76 | 0.003 |
| Treatment x condition | F(2, 165) = 3.36 | 0.04 | 0.039 |
| Treatment x Perceived gender x condition | F(2, 165) = 0.75 | 0.48 | 0.009 |

Table notes: A total of 94 subjects thought the applied touch stimulations were administered by a male masseur (N = 77 thought by a female) but they did not know all touch stimulations were in fact always from the same male masseur.

**Supplementary file 1. Table 1D.** Treatment effects on physiological indices

| ***Source*** | ***F*** | ***p*** | ***η_p_^2^*** | ***Post-hoc Bonferroni***  ***corrected tests*** |
| --- | --- | --- | --- | --- |
| *Heart rate (n = 168)* |  |  | | |
| Condition | F(3,495)=16.72 | < 0.001 | 0.092 | Higher heart rate following gentle stroking touch than medium massage |
| Treatment | F(2, 165) = 0.50 | 0.61 | 0.006 | *-* |
| Condition x treatment | F(6, 495) = 0.70 | 0.65 | 0.008 | *-* |
| *HRV-HF (n = 168)* |  | | | |
| Condition | F(3,495)=12.66 | < 0.001 | 0.071 | Higher HF-HRV following both gentle touch and medium massage compared with rest intervals (*p*s < 0.002) and following medium pressure massage than gentle stroking touch (*p* = 0.006) |
| Treatment | F(2, 165) = 0.70 | 0.50 | 0.008 | *-* |
| Condition x treatment | F(6, 495) = 0.40 | 0.88 | 0.005 | *-* |
| *HRV-DFAα1 (n = 168)* |  |  | | |
| Condition | F(3,495)=12.66 | < 0.001 | 0.071 | Reduced DFA-a1 reflecting higher HRV following both the gentle touch and medium massage compared with rest intervals (*p*s < 0.022) and during medium pressure massage relative to gentle stroking touch (*p* = 0.002) |
| Treatment | F(2, 165) = 0.68 | 0.51 | 0.008 | *-* |
| Condition x treatment | F(6, 495) = 0.33 | 0.92 | 0.004 | *-* |
| *SCR (n = 165)* |  |  | | |
| Condition | F(3, 162) = 85.01 | < 0.001 | 0.344 | Increased SCR amplitude for both gentle touch and pressure massage than during rest intervals (*p*s < 0.001) and during medium pressure massage than gentle stroking touch (*p* < 0.001) |
| Treatment | F(2, 162) = 0.95 | 0.39 | 0.012 | *-* |
| Condition x treatment | F(6, 486) = 0.43 | 0.86 | 0.005 | *-* |

Table notes: ECG data of 3 subjects were not recorded correctly. 6 subjects were excluded from the SCR analysis due to the failure of recording and low-quality data.
